# The recurrent *SETBP1* c.2608G > A, p.(Gly870Ser) variant in a patient with Schinzel-Giedion syndrome: an illustrative case of the utility of whole exome sequencing in a critically ill neonate

**DOI:** 10.1186/s13052-020-00839-y

**Published:** 2020-05-27

**Authors:** Maria Pia Leone, Pietro Palumbo, Orazio Palumbo, Ester Di Muro, Massimiliano Chetta, Nicola Laforgia, Nicoletta Resta, Alessandro Stella, Stefano Castellana, Tommaso Mazza, Marco Castori, Massimo Carella, Nenad Bukvic

**Affiliations:** 1 Division of Medical Genetics, Fondazione IRCCS Casa Sollievo della Sofferenza, San Giovanni Rotondo, FG Italy; 2 Division of Medical and Laboratory Genetics, Azienda Ospedaliera di Rilievo Nazionale “Antonio Cardarelli”, Naples, Italy; 3 Division of Neonatology and Neonatal Intensive Care, Azienda Ospedaliero-Universitaria Consorziale Policlinico di Bari, Bari, Italy; 4 Medical Genetics, Department of Biomedical Sciences and Human Oncology (DIMO), University of Bari “Aldo Moro”, Bari, Italy; 5 Bioinformatics Unit, Fondazione IRCCS Casa Sollievo della Sofferenza, San Giovanni Rotondo, FG Italy; 6 Division of Medical Genetics, Azienda Ospedaliero-Universitaria Consorziale Policlinico di Bari, Bari, Italy

**Keywords:** Schinzel-Giedion syndrome, Whole exome sequencing, *SETBP1*, Critically ill neonate

## Abstract

**Background:**

Schinzel-Giedion syndrome (SGS) is a multiple malformation syndrome mainly characterized by severe intellectual disability, distinctive facial features, and multiple congenital anomalies, including skeletal abnormalities, genitourinary and renal malformations, cardiac defects, as well as an increased pediatric cancer risk. Recently, SGS has been associated with de novo heterozygous deleterious variants in the *SETBP1* gene; to date, nine different variants, clustering in exon 4 of *SETBP1*, have been identified in 25 patients.

**Case presentation:**

In this study, by using Whole Exome Sequencing (WES), we identified a patient with a recurrent missense mutation in *SETBP1*, the c.2608G > A, p.(Gly870Ser) variant, previously reported as likely pathogenic. This finding allowed us to confirm the suspected clinical diagnosis of SGS. Clinical features of patients carrying the same variant, including our patient, were evaluated by a review of medical records.

**Conclusions:**

Our study confirms SGS as a severe disorder potentially presenting at birth as a critically ill neonate and demonstrates the causal role of the c.2608G > A, p.(Gly870Ser) variant in the etiology of the syndrome. Moreover, although the cohort of *SETBP1*-patients reported in the literature is still small, our study reports for the first time the prevalence of the variant (about 27%, 7/26). Finally, given the heterogeneity of clinical presentations of affected patients hospitalized in Neonatal Intensive Care Units (NICU) and/or Pediatric Intensive Care Units (PICU), in agreement with emerging data from the literature, we suggest that WES should be used in the diagnosis of unexplained syndromic conditions, and even as part of a standard first-line diagnostic approach, as it would allow a better diagnosis, counseling and management of affected patients and their families.

## Background

Schinzel-Giedion syndrome (SGS, OMIM 269150) is an ultrarare autosomal dominant disorder characterized by neurodevelopmental disorder, characteristic facial features with midface hypoplasia, cardiac, skeletal and genitourinary malformations, and an increased pediatric cancer risk [1]. The exact frequency of SGS is unknown and only ~ 50 affected individuals have been reported to date. The molecular basis of SGS was resolved in 2010, with the identification of de novo heterozygous variants in the *SETBP1* gene (OMIM 611060) [2]. The *SETBP1* gene is located on chromosome region 18q21.1 and encodes for an oncogene-binding protein which binds to SET domains that are involved in the methylation of lysine residues on histone tails. The multisystemic involvement of SGS is recapitulated by the ubiquitous expression of *SETBP1*, whose biological functions remain not yet fully elucidated [3].

To the best of our knowledge, nine different *SETBP1* deleterious variants have been reported in 25 individuals, and all of these variants cluster in exon 4, within a hotspot of 12 base pairs corresponding to aminoacids 867 to 871 of the encoded protein [1, 4–13]. The affected protein region is highly conserved and is a peptide sequence crucial for the degradation of the protein itself. The SGS-specific variants increase protein stability by interfering with ubiquitination and lead to the accumulation of the protein in the cell [14]. Interestingly, some of the *SETBP1* variants which cause SGS when inherited, have been repeatedly registered as somatic events in several types of myeloid malignancies [15–18].

In addition to point mutations, two additional patients with a proximal interstitial 18q microdeletion selectively involving *SETBP1*, presenting with milder phenotypes apparently distinct from the full-blown SGS [19] have been described. Finally, also a subject with the *SETBP1* frameshift p.(Asn395Lysfs*2) variant and dysmorphic features, coordination deficits in fine motor skills and behavioral problems has been described. Neurological evaluation showed no hypotonia; no abnormal findings were observed on genital examination. Hearing and vision were normal. Urinary tract ultrasound and brain magnetic resonance imaging were normal [20]. These cases testify that *SETBP1* null alleles cause a less severe phenotype and that the point variants more typical for SGS are likely to act with a dominant negative effect.

Here, we report the case of a critically ill neonate presenting to the Neonatal Intensive Care Unit with multiple malformations and subsequently diagnosed with SGS. The diagnosis was reached by performing the Exome Sequencing, which revealed the presence of the *SETBP1* recurrent c.2608G > A, p.(Gly870Ser) variant. We also compared the phenotypic data of our patient with the phenotypic data reported so far in the literature, which were collected from other patients carrying the same variant in the *SETBP1* gene. Finally, we discuss the utility of WES in establishing an etiological basis in clinically and genetically heterogeneous conditions, which are often found in affected patients hospitalized in Neonatal Intensive Care Units (NICU) and/or Pediatric Intensive Care Units (PICU).

## Case report

The proband is a Caucasian 4-day-old neonate. A clinical genetics assessment for a multisystemic disease -which was not recognized antenatally- was carried out at the NICU. He was the third male child of a healthy non-consanguineous couple. At the time of his birth, his father and mother were 42 and 36 years old, respectively. Family history was unremarkable, especially for neurodevelopmental disorders, brain abnormalities, recurrent miscarriages, other birth defects and/or genetic illnesses. The child was born after an uneventful full-term gestation. Birth weight was 2.630 g (3rd centile), length 47 cm (4th centile), head circumference 32 cm (2nd centile), and APGAR scores 8/8 at 1′/5′, respectively.

**Fig. 1:**
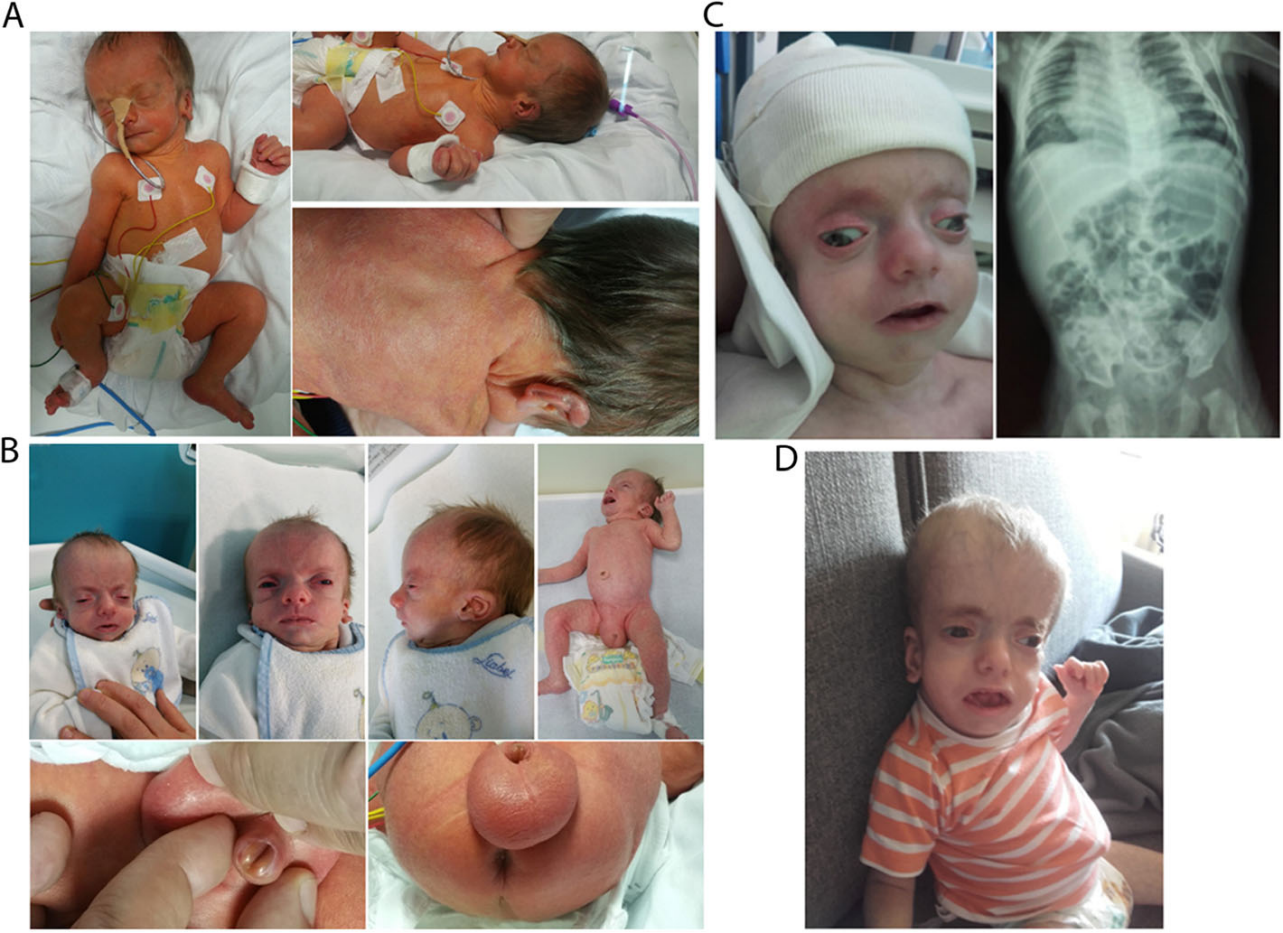
The main clinical features observed in the patient at 4 days (**a**), 21 days (**b**), 4 months (**c**) and 6 months (**d**). Detailed clinical description is reported in the text

Admission to the Neonatal Intensive Care Unit was requested for the combination of heart murmur, external genitalia anomalies and hypotonia, and respiratory distress. Physical examination on day 4 revealed triangular face, frontal bossing, clinically apparent hypertelorism, periorbital edema, depressed nasal bridge, bulbous nose, anteverted nares, low hanging columella, short philtrum, downturned corners of mouth, thin lips, hypoplastic chin, low set and posteriorly rotated dysmorphic ears, and low posterior hairline with apparent redundant neck skin (Fig. 1a). Ultrasound screening revealed atrial septal defect (ASD), hypoplastic corpus callosum and bilateral pyelectasis. Facial features worsened in the following weeks; in addition, the scrotum was severely enlarged, and penile hypospadias was noted (Fig. 1b). In addition, beside described clinical symptoms, the patient showed poor feeding, irritability, reduced activity and retching due to rapid-onset of hydrocephalus with increased Intracranial Pressure (ICP). For these reasons, he was transferred to another hospital for underwent a urgent surgical treatment consisting in the insertion of a drainage system (shunt), and therefore we lost the possibility of periodical follow up for the next 4 months. However, the parents continued to keep us informed. No significant abnormalities were detected when blood tests were performed, including complete blood cell count, blood gas analysis, conventional biochemical test, thyroid function test, amino acid analysis, and lysosomal enzyme activity analysis. The results of urinary organic acid and uronic acid analyses were also normal. In the clinical documentation sent by the parents, we found: RX examination with dorsal levoscoliosis, broad ribs and relatively long clavicles (Fig. 1c). Results of rapid standard karyotyping revealed a normal male (46,XY), while high resolution SNP-array analysis revealed a 0,6 Mb microduplication of uncertain clinical significance on the chromosome region 10q22.3, inherited from his healthy father (arr[hg19]10q22.3(81,362,685 × 2,81,385,244-81,986,097 × 3,81,990,582 × 2)pat). Even though the objective examinations were suggestive for Schinzel-Giedion Syndrome (Fig. 1d), we had to take into consideration all possible differential diagnoses and, for this reason, Whole Exome Sequencing Analysis was performed.

## Materials and methods

### Whole exome sequencing (WES)

The patient’s parents provided written informed consent to molecular testing and to the full content of this publication. This study was performed in accordance with the Declaration of Helsinki (1984) and its subsequent revisions. Genomic DNA was extracted from peripheral blood samples using the Bio Robot EZ1 (Quiagen, Solna, Sweden). DNA quantity and quality were measured by NanoDrop 2000 C Spectrophotometer (Thermo Fisher Scientific, Waltham, MA, USA). The sample was then quantified with the Qubit fluorometer (Thermo Fisher Scientific, Waltham, MA, USA) using the Quant-iT dsDNA BR Assay kit (Thermo Fisher Scientific, Waltham, MA, USA), according to the manufacturer’s instructions.

The exome of the index patient and his parents was enriched using the SureSelect^QXT^ Target Enrichement system (Agilent Technologies, Santa Clara, CA, USA), according to the manufacturer’s instructions. The libraries were then sequenced on the NextSeq500 Sequencing System (Illumina Inc., San Diego, CA, USA), using a High Output 300 cycles flow cell (Illumina Inc., San Diego, CA, USA).

Quality of the sequenced sequences was checked using the FastQC software *[S. Andrews, FastQC: a Quality Control Tool for High Throughput Sequence Data, 2010 available online at:**http://www.bioinformatics.babraham.ac.uk/projects/fastqc**]* and then aligned against the hg19 human reference genome by the BWAmem [21]. Depth of coverage analysis was carried out through the TEQC R Package on the produced *.bam file, thus obtaining measures of sample and region-specific sequencing coverage [22]. The identification of single nucleotide variants and small insertions/deletions was performed using GATK ver. 3.7 [23]. Variants were functionally annotated through the Annovar Software [24], with the Ncbi Human RefSeq as transcript reference system [25]. Annotated variants were also checked for novelty in public collections, such as dbSNP ver. 151 [26], ExAC and gnomAD [27]. Furthermore, prediction of functional effect of non-synonymous and splice site variants were retrieved by the dbNSFP v3.4 database [28].

Subsequently, the prioritization of the variants started excluding those described as benign and likely benign. Then the remaining variants which passed this filtering were classified on the basis of their clinical relevance as pathogenic, likely pathogenic or variant of uncertain significance by using the following criteria: (i) nonsense/frameshift variant in genes previously described as disease-causing by haploinsufficiency or loss-of-function; (ii) missense variant located in a critical or functional domain; (iii) variant affecting canonical splicing sites (i.e., ±1 or ± 2 positions); (iv) variant absent in allele frequency population databases; (v) variant reported in allele frequency population databases, but with a minor allele frequency (MAF) significantly lower than expected for the disease; (vi) variant predicted and/or annotated as pathogenic/deleterious in ClinVar and/or LOVD.

The putative pathogenic variants identified following this pipeline were confirmed by direct Sanger sequencing, and the segregation analysis was carried out. Sanger sequencing was performed using BigDye Terminator v3.1 Cycle Sequencing kit (Applied Biosystems, Foster City, CA, Stati Uniti) on 3130xl Genetic Analyzer Sequencer (Applied Biosystems, Foster City, CA, Stati Uniti). The clinical significance of the identified putative variants was interpreted according to the American College of Medical Genetics and Genomics (ACMG) [29].

## Results

More than 15 million paired-end sequences were produced for this sample with a mean base quality score of 31 (i.e., probability of being an erroneous call less than 1/1000) and a 64X mean depth of coverage.

**Fig. 2:**
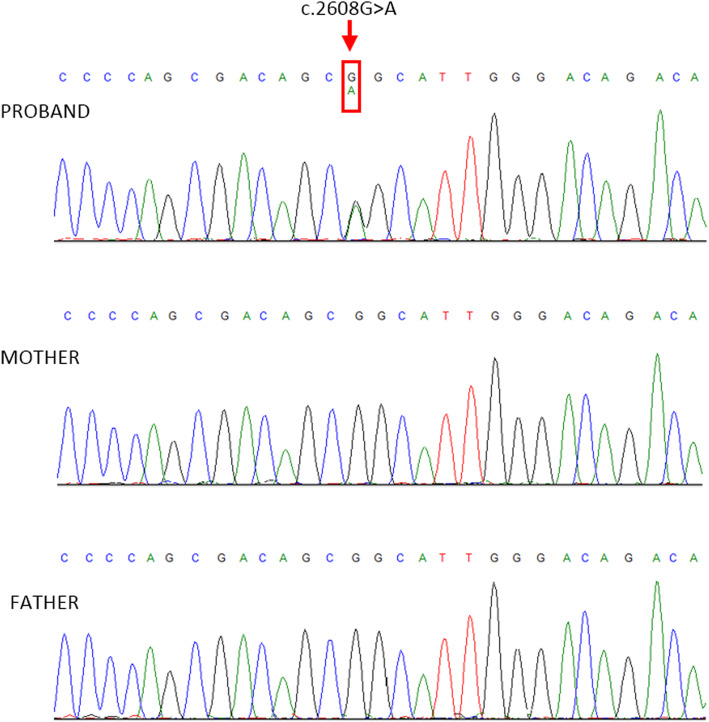
Genomic DNA sequence of the *SETBP1* gene in the proband and his parents. The patient exhibited a missense mutation, c.2608G > A, indicated by red arrow, whereas his unaffected parents carried the wild-type allele

NGS analysis of the proband revealed that he was heterozygous for a de novo, previously described, mutation in *SETBP1* (NM_015559) c.2608G > A, p.(Gly870Ser). The detected variant, already reported in dbSNP (rs267607040), was detected with a > 100X read depth (60 out of 159 reads supporting the alternate allele) and an elevated quality score. The identified candidate disease-causing variant was confirmed by direct Sanger Sequencing using specific primers (*SETBP1*, exon 4, Forward Primer: 5′- AGACTGATGGCCAACTCCC; Reverse Primer: 5′- GTTCTTTGTGCTGGTGTCGG) (Fig. 2).

**Fig. 3:**
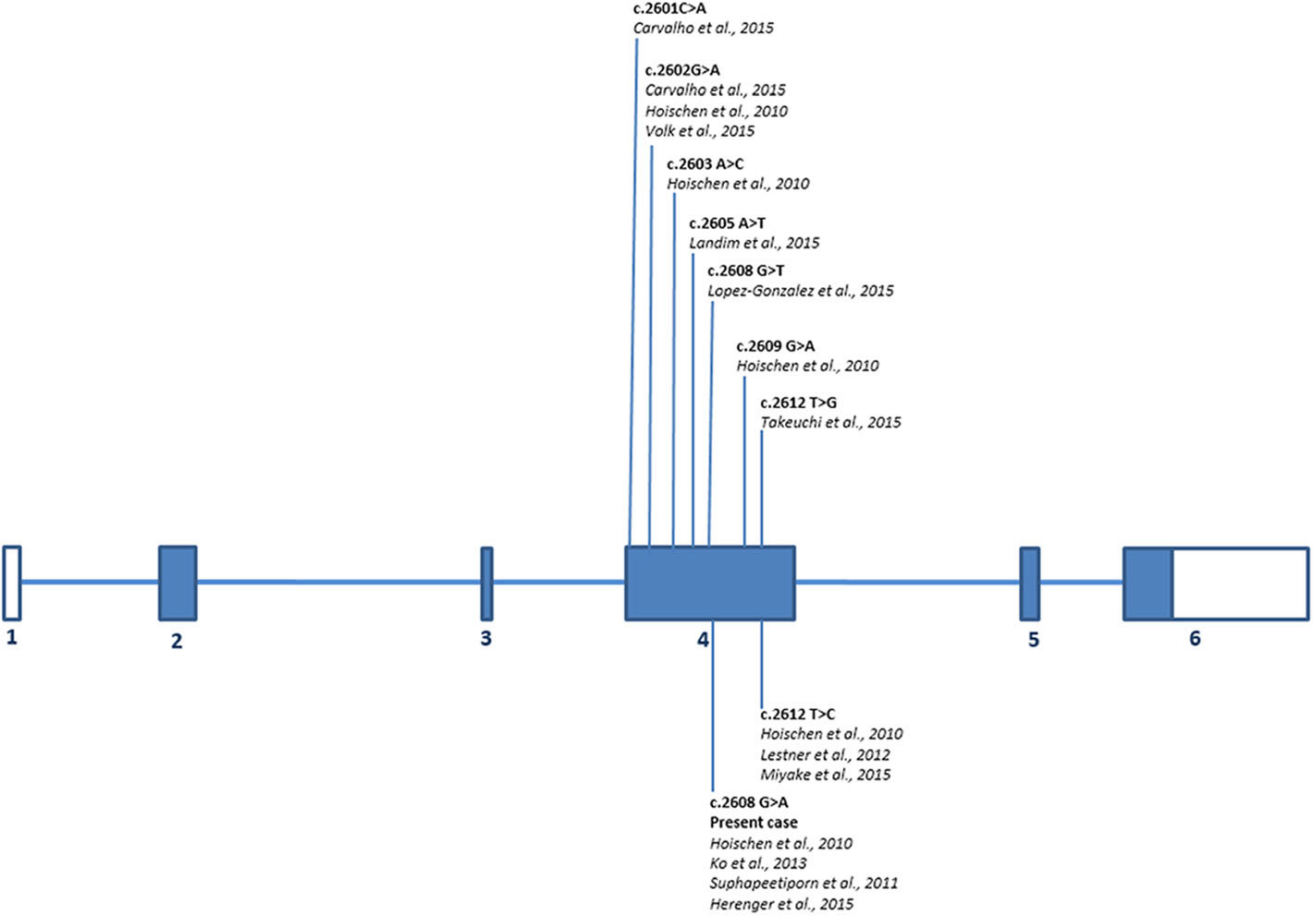
Schematic representation of the *SETBP1* with positions of all known deleterious variants published to date and include the present one

In the literature, the frequency of the variant is reported to be very low in the ExAC global population (MAF: 0.00002) and absent in the European population. The variant affects a residue that is highly conserved in different species of vertebrates (Fig. 3) [30], and, according to the American College of Medical Genetics and Genomics guidelines [29], the variant was classified as pathogenetic. For the nucleotide variants nomenclature we used the format recommended by the Human Genome Variation Society (HGVS, http://www.hgvs.org) recommendations and reported in the Leiden Open Variation Databases (LOVD) (https://databases.lovd.nl/shared/individuals/00289344).

Bioinformatic analysis excluded any other pathogenic or likely pathogenic heterozygous variants or biallelic variants in genes associated with compatible phenotypes. Given this result, the overall phenotype of the neonate was reviewed and, eventually, the molecular suspect of SGS was confirmed after a focused examination of the clinical findings.

## Discussion and conclusions

Here, we report the case of a neonate suffering from SGS and carrier of the de novo recurrent variant c.2608 G > A, p (Gly870Ser) in *SETBP1* (NM_015559). The *SETBP1* gene encodes a protein which contains a SKI-homology domain, a SET-binding region which adds to three nuclear localization signals. Oakley and his collaborators [31] demonstrated that, by transducing murine bone marrow progenitors with high titer retrovirus expressing SETBP1, SETBP1 is able to bind to Hoxa9/10 promoters upregulating the two genes. In line with these findings, Piazza and his collaborators focused on the potential role of *SETBP1* as a transcriptional modulator and showed that SETBP1 interacts with gDNA through its AT-hook domains by forming a multiprotein complex including HCF1, KMT2A, PHF8, and PHF6, which increases chromatin accessibility. Gene ontology analysis of deregulated SETBP1 target genes indicates that they are key controllers of visceral organ development and brain morphogenesis [32].

**Fig. 4:**
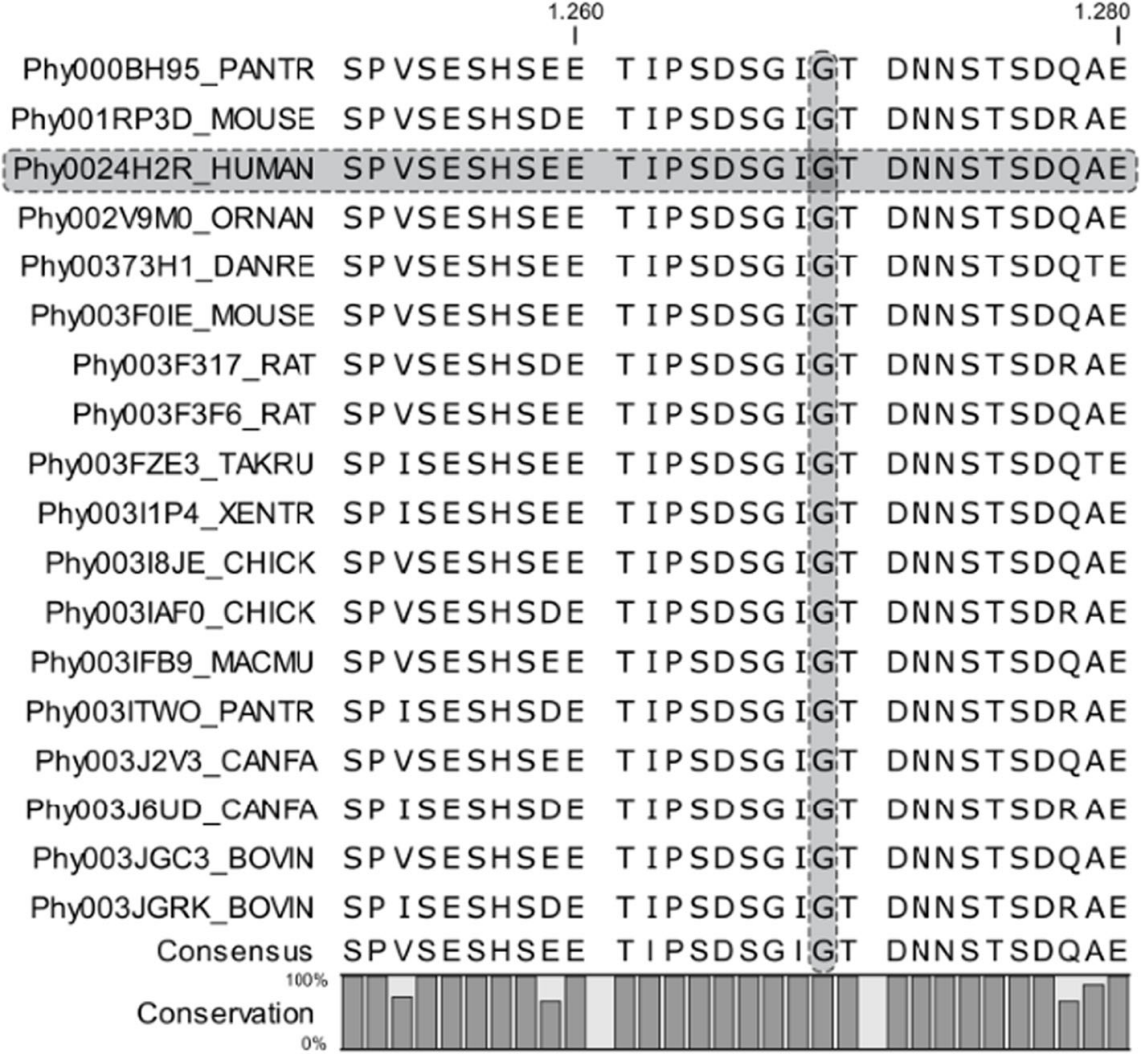
Sequence alignment for SETBP1 homologous sequences (from 1251 to 1280 alignment site). Human sequence (“Phy0024H2R_HUMAN”) and investigated amino acid site (protein position: 870; alignment position: 1269) evidenced in grey. Data retrieved from Phylome 533, PhylomeDB ver.4

To the best of our knowledge, nine different point mutations have been reported in 26 SGS patients, including our patient, and all of them occurred within a hotspot of 12 base pairs in exon 4 of the *SETBP1* gene (chromosome position: 42531904–42,531,918), corresponding to aminoacids 867 to 871 of the encoded protein. The nine variants to date identified are listed in Table 1 and depicted in Fig. 3. This recurrently mutated region, localized in the SKI homology region of the protein, is highly conserved in vertebrates (Fig. 4), suggesting that it might have an important biological role. It has been identified as a specific sequence that pilots the protein to the initial degradation step. It contains a consensus-binding region for β-TrCP1, the substrate recognition subunit of the E3 ubiquitin ligase, and might be critical for protein degradation through ubiquitin binding. When mutated, SETBP1 protein fails to bind to this E3 ligase subunit, and protein stability increases by interfering with ubiquitination, leading to the accumulation of SETBP1 protein in the cells [15, 32]. The disease causing the mutation identified in our patient had been previously reported and classified as likely pathogenetic in 6 of the 25 patients with molecularly confirmed SGS [2]. For this reason, from a molecular point of view, our report is useful to allow the definitive classification of this variant as pathogenetic. Interesting to note, although patients with a clinical diagnosis of SGS carrying a pathogenetic variant in *SETBP1* are still few, the c.2608G > A, p.(Gly870Ser) variant is, together with the c.2612 T > C, p.I871T, the most frequent one with a prevalence of about 27% (7/26, Table 1).

**Table 1:** Point mutations in *SETBP1* gene reported in 26 patients with a clinical diagnosis of Schinzel-Giedion Syndrome

Patients reported	Mutation site								
	p.S867R	p.D868N	p.D868A	p.S869C	p.G870C	p.G870D	p.G870S	p.I871S	p.I871T
Present case							*n* = 1		
*Carvalho* et al.*, 2015* [6]	*n* = 1	*n* = 1							
*Hoischen* et al.*, 2010* [2]		*n* = 4	*n* = 1			*n* = 1	*n* = 1		*n* = 5
*Volk* et al.*, 2015* [13]		*n* = 1							
*Landim* et al.*, 2015* [10]				*n* = 1					
*Ko* et al.*, 2013* [4]							*n* = 1		
*Suphapeetiporn* et al.*, 2011* [5]							*n* = 2		
*Herenger* et al.*, 2015* [9]							*n* = 2		
*Lopez-Gonzalez* et al.*, 2015* [7]					*n* = 1				
*Lestner* et al.*, 2012* [8]									*n* = 1
*Miyake* et al.*, 2015* [11]									*n* = 1
*Takeuchi* et al.*, 2015* [12]								*n* = 1

We limited our literature review to all patients carrying the c.2608G > A, p.(Gly870Ser). The seven patients, including our patient, to date identified are listed in Table 2. The clinical comparison shows that the emerging phenotype remains comparable, despite a lack of clinical information in some reports (Table 2). In detail, the patients present large anterior fontanelle (6/7), prominent forehead/frontal bossing (7/7), midface hypoplasia/retraction (7/7), ocular hypertelorism (5/7), short and upturned nose (7/7) and low-set ear(s) (6/7). Other hallmarks of SGS syndrome, which are important clues for the diagnosis, are skeletal system malformations (7/7) in the form of synchondrosis, clinodactyly and camptodactyly, short first metacarpal bones, bowing of both tibiae, talipes and deformities of both feet, long bones and broad ribs, and urogenital and renal defects (6/7) in the form of hydronephrosis, micropenia and hypospadias. Neurological problems are also present, such as severe intellectual disability (7/7), seizures (7/7), hearing impairment (6/7), ventriculomegaly (6/7), and vision impairment (4/7). Other impairments observed since birth are characterized by feeding and respiratory problems, often associated with epileptic seizures (our patient and #4, Table 2), and neurodegenerative process caused by deficient white matter myelination and abnormal pattern of myelination (patient 2, Table 2), which is emerging as an important, but under-reported, central nervous system findings in patients with SGS [12, 33]. Accordingly, the overall clinical picture of the seven individuals carrying the c.2608 G > A, p (Gly870Ser) variant is homogeneous without significant differences from the remaining patients with SGS reported in medical literature. This clinical comparison allowed us to confirm that SGS is a severe disorder potentially presenting at birth (critically ill neonate). The molecular basis of SGS remains homogeneous with an apparently strict association between recognizable phenotype and mutation site(s).

**Table 2:** Major clinical findings reported in patients with c.2608 G > A, p (Gly870Ser) mutation in *SETBP1* gene

	Present case	*patient 1* *(Hoischen* et al.*, 2010* [2]*)*	*patient 2* *(Suphapeetiporn* et al.*, 2011* [5]*)*	*patient 3* *(Suphapeetiporn* et al.*, 2011* [5]*)*	*patient 4* *(Ko* et al.*, 2013* [4]*)*	*patient 5* *(Herenger* et al.*, 2015* [9]*)*	*patient 6* *(Herenger* et al.*, 2015* [9]*)*
Gender	*Male*	*Female*	*Male*	*Male*	*Male*	*Male*	*Male*
Age	*4-day-old*	NA	*1-month-old*	*1-month-old*	*3-month-old*	*6-years-old*	*15-years-old*
Developmental delay	+	+	+	+	+	+	+
Seizures	+	+	+	+	+	+	+
Vision impairment	–	+	–	NA	+	+	+
Ventriculomegaly	+	NA	+	+	+	+	+
Abnormal pattern of myelination	–	–	+	–	–	–	–
Hearing impairment	+	+	+	NA	+	+	+
Typical craniofacial features	+	+	+	+	+	+	+
Genital anomalies	+	+	+	+	+	+	+
Hydronephrosis or vesicoureteral reflux	–	+	+	+	+	+	+
Cardiac defect	+	+	NA	–	–	–	–
Characteristic skeletal malformation	+	+	+	+	+	+	+
Feeding problems	+	–	–	–	+	–	–

+ = feature present; − = feature absent; NA = feature not reported

More importantly, in our case, the molecular diagnosis of SGS was made before a definitive clinical diagnosis and it was possible by performing the Whole Exome Sequencing on samples obtained shortly after birth and rapid cytogenomic screening. The utility of fast exome/genome sequencing on critically ill neonates is startig to be included in the current clinical genomics applications [34]. Literature data are heterogeneous and, therefore, preliminary, but the diagnostic yield of such an approach is strongly influenced by the selection strategy of patients. For example, while a randomized study on unselected cases of patients referred to Neonatal Intensive Care Units had a positive rate of only 1 out of 29 [35], another study on 50 neonates selected with a phenotype-driven approach allowed a molecular diagnosis in 29 (54%) [36]. In our practice, we are still working on the appropriate, evidence-based approach of introducing genomic analysis in the routinary clinical activities of Neonatal Intensive Care Units for critically ill neonates. However, our experience shows that a phenotype-driven approach carried out by clinical geneticists working in a team with neonatologists and child neurologist should be preferred as it eases the post-analytical phase for a timely production of the written results. In our patient, although the facial gestalt at birth was evocative of a multiple malformation syndrome, the molecular results were crucial in establishing the diagnosis due to the presumably evolving nature of SGS. In our patient, the diagnosis of SGS allowed clinicians to (i) correctly focus their attention on borderline features, such as craniofacial anomalies which could have raised the suspicion of an underlying genetic neurological or metabolic disease, (ii) put the patient in a follow-up program for monitoring the syndrome-specific cancer risk, and (iii) provide the family with precise information for future family planning and prenatal diagnosis.

In conclusion, our case confirms that SGS is a severe disorder potentially presenting at birth as a critically ill neonate. The clinical comparison of patients carrying the same point mutation c.2608G > A, p.(Gly870Ser) allowed us to establish definitively the pathogenic role of this genetic alteration and, although the cohort of *SETBP1*-patients reported in the literature is still small, to report for the first time the prevalence of the variant, which is about 27% (7/26). Many previous studies showed that WES is a valid option for genetic testing in patients with syndromic manifestations when chromosomal imbalances and other hypothesis with available targeted studies have been ruled out. Considering that the treatment provided in Neonatal Intensive Care Units and/or Pediatric Intensive Care Units is one of the most cost-effective ones in high-cost healthcare, we suggest the use of WES to establish the etiological basis in clinically and genetically heterogeneous conditions of affected patients. However, issues remain on the most reasonable application of this technology in routinary clinical care.

Our experience and emerging evidence from the literature suggest that Whole Exome/Genome diagnostic facilities should only be made accessible to the expert professionals in multidisciplinary teams.

## Data Availability

Not applicable.

## Abbreviations

SGS: Schinzel-Giedion syndrome
WES: Whole Exome Sequencing
NICU: Neonatal Intensive Care Units
PICU: Pediatric Intensive Care Units
ASD: Atrial septal defect
ICP: Intracranial Pressure
MAF: Minor Allele Frequency
ACMG: American College of Medical Genetics and Genomics
HGVS: Human Genome Variation Society
LOVD: Leiden Open Variation Databases
